# Insect morphometry is reproducible under average investigation standards

**DOI:** 10.1002/ece3.7075

**Published:** 2020-12-08

**Authors:** Sándor Csősz, Bernhard Seifert, István Mikó, Brendon E. Boudinot, Marek L. Borowiec, Brian L. Fisher, Matthew Prebus, Jayanthi Puniamoorthy, Jean‐Claude Rakotonirina, Nicole Rasoamanana, Roland Schultz, Carolyn Trietsch, Jonah M. Ulmer, Zoltán Elek

**Affiliations:** ^1^ MTA‐ELTE‐MTM Ecology Research Group Budapest Hungary; ^2^ Evolutionary Ecology Research Group Centre for Ecological Research Institute of Ecology and Botany Vácrátót Hungary; ^3^ Senckenberg Museum of Natural History Görlitz Görlitz Germany; ^4^ Department of Biological Sciences University of New Hampshire Durham NH USA; ^5^ Department of Entomology & Nematology University of California Davis CA USA; ^6^ Department of Entomology, Plant Pathology and Nematology University of Idaho ID USA; ^7^ Department of Entomology California Academy of Sciences San Francisco CA USA; ^8^ Department of Biological Sciences National University of Singapore Singapore; ^9^ Madagascar Biodiversity Center Antananarivo Madagascar; ^10^ Département d'Entomologie Université d'Antananarivo Antananarivo Madagascar; ^11^ Pennsylvania State University University Park PA USA; ^12^ Staatliches Museum für Naturkunde Stuttgart Germany

**Keywords:** entomology, measurement error, morphology, repeatability, species delimitation, taxonomy

## Abstract

1. Morphometric research is being applied to a growing number and variety of organisms. Discoveries achieved via morphometric approaches are often considered highly transferable, in contrast to the tacit and idiosyncratic interpretation of discrete character states. The reliability of morphometric workflows in insect systematics has never been a subject of focused research, but such studies are sorely needed. In this paper, we assess the reproducibility of morphometric studies of ants where the mode of data collection is a shared routine.
2. We compared datasets generated by eleven independent gaugers, that is, collaborators, who measured 21 continuous morphometric traits on the same pool of individuals according to the same protocol. The gaugers possessed a wide range of morphometric skills, had varying expertise among insect groups, and differed in their facility with measuring equipment. We used intraclass correlation coefficients (ICC) to calculate repeatability and reproducibility values (i.e., intra‐ and intergauger agreements), and we performed a multivariate permutational multivariate analysis of variance (PERMANOVA) using the Morosita index of dissimilarity with 9,999 iterations.
3. The calculated average measure of intraclass correlation coefficients of different gaugers ranged from *R* = 0.784 to *R* = 0.9897 and a significant correlation was found between the repeatability and the morphometric skills of gaugers (*p* = 0.016). There was no significant association with the magnification of the equipment in the case of these rather small ants. The intergauger agreement, that is the reproducibility, varied between *R* = 0.872 and *R* = 0.471 (mean *R* = 0.690), but all gaugers arrived at the same two‐species conclusion. A PERMANOVA test revealed no significant gauger effect on species identity (*R* ^2^ = 0.69, *p* = 0.58).
4. Our findings show that morphometric studies are reproducible when observers follow the standard protocol; hence, morphometric findings are widely transferable and will remain a valuable data source for alpha taxonomy.

## Graphical abstract

This paper reveals that morphometric findings are reliable and widely transferable in insect research and can represent a valuable data source in the field of ecological and evolutionary studies. The manuscript can also help to educate readers on important issues regarding measurements and measurement error. This is especially important in the field of ecology and systematics today because there is a general lack of formal training in these important concepts in the 21st century biological science curriculum.

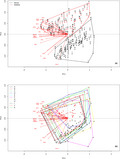

## 1 INTRODUCTION

The phenotype of organisms varies continuously during development and through evolutionary time. Continuous morphological variation is captured for numerous purposes in the life sciences via the practice of morphometry: the measurement of the size and shape of anatomical forms. Morphometry has yielded novel findings in evolution (Esquerré et al., 2020) and has been used to assess fluctuating asymmetry (Palmer, 1993; Klingenberg, 2015), ontogeny (Csősz & Majoros, 2009; Shingleton et al., 2007), ecomorphism (Anderson et al., 2019; Mahendiran et al., 2018; Tomiya & Meachen, 2018), and in human clinical practice (Bartlett & Frost, 2008). Among other applications, morphometric data are also key for alpha taxonomy, the discipline of formally differentiating and describing species and higher taxa. This is exemplified by the development of phenetics in the twentieth century (Michener & Sokal, 1957; Sokal & Sneath, 1963) and by numerous modern studies in other frameworks, such as for plants (Chuanromanee et al., 2019; Savriama, 2018), animals (Inäbnit et al., 2019; Villemant et al., 2007), and other organisms (Fodor et al., 2015; McMullin et al., 2018). Continuous data are also valuable, for modeling evolutionary histories (e.g., Parins‐Fukuchi, 2017, 2020). Thus, the morphometric approach constitutes a fundamental and crucial practice for the study of phenotypes in biodiversity research.

Morphology is traditionally considered to comprise both continuous and discrete traits (Artistotle, 350; Remane, 1952; Rensch, 1947; Thompson, 1917). Discrete states were established as the basic comparative units in animal alpha taxonomy from its formalization (Linnaeus, 1758) and have become a key means of scoring data for phylogenetic analysis, particularly after Hennig (1950, 1966). The reproducibility of scoring discrete states is an issue; however, as qualitative perception of phenotype not only requires specific training and considerable experience but can also be plagued by arbitrariness (Bond & Beamer, 2006), meaning that variation may simply come from individual (mis‐)interpretation. The qualitative approach commonly uses verbal species descriptions that are often subjective or difficult to articulate. Therefore, information transfer, if at all reliable, is based on one‐to‐one knowledge sharing mechanisms, and requires logically structured linguistic hierarchies such as the Hymenoptera Anatomy Ontology (Yoder et al., 2010).

In contrast to this relatively idiosyncratic approach, morphometry is considered transferable. It converts variation in the shape and size of anatomical traits, and number and arrangement of anatomical elements into numerical values, allowing for the dissemination of reproducible, phenotype‐based knowledge. Today, an increasing number of morphology‐based insect alpha‐taxonomists use morphometric data and provide numeric keys to species (Steiner et al., 2006; Csősz et al., 2015; Seifert, 2018). If observers arrive at the same conclusion by measuring traits according to the same protocol, findings are believed to be reliable and transferable. If one can measure a trait, anyone else should be able to reproduce it.

All measurements are subject to error, however. Agreement among different observers and within a single observer's measurements are affected by a number of sources, such as the skills of the observer (if human input is required), the precision and accuracy of the equipment, clear interpretation and appropriate understanding of the character recording protocol, and other parameters. All of the uncertainty factors mentioned above are common in practice, and the fact that it is impossible to control every source of measurement variation challenges morphometry‐based research (Wolak et al., 2012). Understanding of the degree to which measurement errors may affect the transferability of findings is urgently needed. During the last few decades, reproducibility issues have been studied in vertebrate systematics (e.g., Oxnard, 1983; Corrucini, 1988; Yezerinac et al., 1992; Helm & Albrecht, 2000; Takacs et al., 2016; Fox et al., 2020), clinical research (e.g., Bland & Altman, 1986; Ridgway et al., 2008; Phexell et al., 2019), social science (e.g., Salganik et al., 2020), molecular phylogeny and genetic clustering (e.g., Huelsenbeck, 1998; Jones et al., 1998; DeBiasse & Ryan, 2019), and morphometric data generally (Andrew et al., 2015). However, to date, reproducibility assessments of morphometric data in entomology are extremely limited (Johnson et al., 2013; Mutanen & Pretorius, 2007).

In order to address the question “to what extent is insect morphometry reproducible?,” we compiled a broad database of morphometric data and performed robust statistical analyses. We used ants, a group in which the application of morphometric data has a long tradition as a model organism (e.g., Brian & Brian, 1949; Brown, 1943). Morphometry has been employed widely in recent myrmecological studies (e.g., Ward, 1999; Baroni Urbani, 1998; Seifert, 1992, 2003; Csősz et al., 2015; Wagner et al., 2017) as the primary method of interpreting anatomical forms and their variation. To evaluate reproducibity, we asked eleven participants to perform repeated measurements on the same set of ant specimens, using the same protocol, and with their own equipment. These participants, or gaugers, were from three continents and six countries, were of diverse levels of skill and expertise, and work with different taxonomic routines. The wide range of morphometric skills and the quality of microscopes used provided us with an overview of the level of reproducibility of morphometric interpretation as it works in daily practice. Our findings are a first step in exploring the reproducibility of morphometric data across entomology.

**Terminology**

**FIGURE 1:**
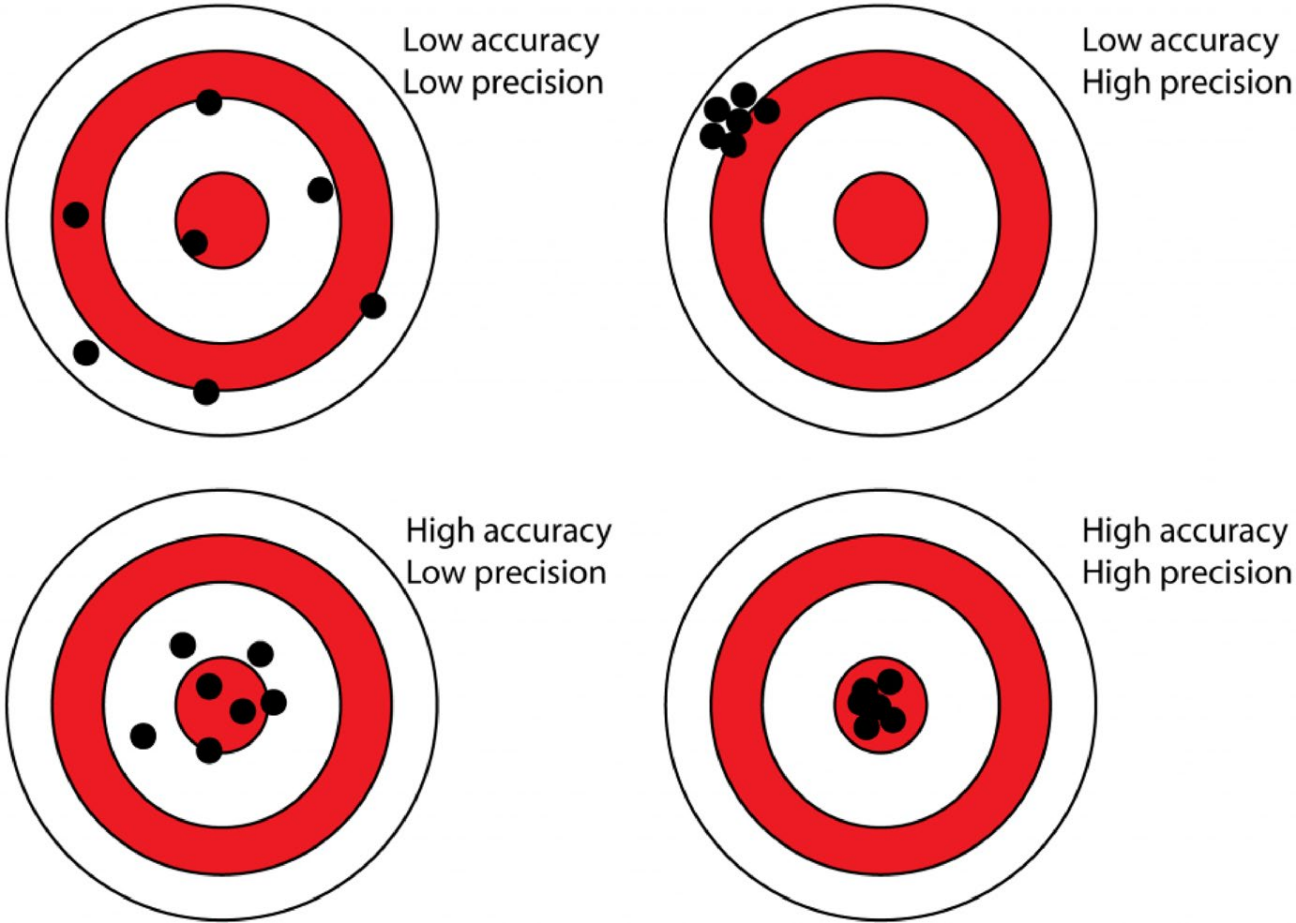
Precision versus accuracy. The bullseye represents the value of the measurand. Accuracy is indicated by closeness to the bullseye—measurements closer to the bullseye are more accurate. Precise measurements are tightly clustered. Accurate and precise measurements are tightly clustered in the bullseye. Graphics produced and used with permission from Dr. Bethan Davies (antarcticglaciers.org)

A number of terms (e.g., “accuracy,” “precision,” “reliability,” “repeatability,” and “reproducibility”) commonly used in association with repeatability studies are defined differently in the literature. To increase the fluency of scientific discourse, we propose to adopt the standard terminology of the National Institute for Standards and Technology (NIST, Taylor & Kuyatt, 2001) of the United States and terms proposed by (Bartlett & Frost, 2008) in biological systematics:

● Accuracy describes the average closeness of the measurement(s) to the value of the measurand (=subject or quantity to be measured) (Figure 1). Accuracy is affected by systematic and random error. We follow the terminology proposed by the NIST in using the phrase "the value of the measurand" instead of the often‐applied "true value of the measurand" (or "a true value") (Taylor & Kuyatt, 2001).

● Precision refers to the closeness of the measurements between pairs of measurements made on the same measurand and applying the same protocol. Precise measurements are tightly clustered, but are not necessarily accurate, that is, close to the value of the measurand (Figure 1). Precision is affected by random error.

● Reliability refers to the amount of measurement error that occurs between observed measurements compared to the inherent amount of variability that occurs between measurands (Bartlett & Frost, 2008).

● Repeatability refers to the degree of agreement between repeat measurements made on the same measurand under the same conditions, that is, made by the same observer, using the same microscope, following the same measurement protocol (Taylor & Kuyatt, 2001). Repeatability can be assessed via intraclass correlation (ICC, see Lessells & Boag, 1987).

● Reproducibility refers to the degree of agreement between measurements made on the same measurand under changing conditions, such as changing principle, method of measurement, observer, instrument, etc. (Taylor & Kuyatt, 2001).

**Sources of errors**

Recognized sources of error in morphometry include three broad classes of observational errors:

1. Random errors, which occur irregularly and hence are unpredictable. Such errors arise in three different ways: random oscillations of the apparatus, mechanical vibrations, and minor positional changes of the subject at every single measurement. This type of error results in dissimilar outcomes, which can be detected by replicated observations. Random error primarily affects precision.

2. Systematic errors, which can be subdivided into (a) observational error, which arises from an individual's bias, unclear description of measuring procedures, lack of proper setting of the equipment, or false data recording due to parallax errors (Seifert, 2002); (b) instrumental error caused by factors such as imperfect calibration, etc., and (c) environmental error that can be ascribed to the effects of the external conditions on the measurements, for example, temperature, illumination, etc. Systematic errors primarily influence a measurement's accuracy, but these sources are predictable.

3. Gross errors, arising from false readings, mistakes in recording data by an observer (e.g., reading or recording 88 instead of 38), or mistakenly set magnification. This type of error seriously affects both precision and accuracy. This source of error can be eliminated by careful reading or recording. This type of error can also be recognized post hoc via comparing the repeated measurements in a pairwise matrix scatterplot (Baur & Leuenberger, 2011).

## 2 MATERIALS AND METHODS

### 2.1 The research objects

As an ideal stress‐test basis for evaluating repeatability of morphometric studies in insect systematic research, we selected ten specimens each of a cryptic species pair, *Nesomyrmex devius* (Csősz & Fisher, 2016) and *Nesomyrmex hirtellus* (Csősz & Fisher, 2016), for a total of twenty ant specimens. Every trait under observation shows overlapping ranges (Seifert, 2009); thus, these species can be classified in multivariate fashion only. Today, cryptic species pairs are considered the most difficult cases and pose extraordinary challenges to systematic biology.

The material is deposited in the California Academy of Sciences, San Francisco, California, U.S.A. The full list of material morphometrically examined in this work is listed in Table S1 (available on Dryad at https://doi.org/10.5061/dryad.q83bk3jfq). Because two specimens suffered a certain degree of damage during the projects due to consecutive postal shipments, making the subsequent gaugers unable to measure them, final analyses were done on only 18 individuals. The ant specimens used in this study comply with the regulations for export and exchange of research samples outlined in the Convention on Biological Diversity and the Convention on International Trade in Endangered Species of Wild Fauna and Flora. For fieldwork conducted in Madagascar, permits to research, collect, and export ants were obtained from the Ministry of Environment and Forest as part of an ongoing collaboration between the California Academy of Sciences and the Ministry of Environment and Forest, Madagascar National Parks and Parc Botanique et Zoologique de Tsimbazaza (Approval Numbers: N° 0142N/EA03/MG02, N° 340N‐EV10/MG04, N° 69 du 07/04/06, N° 065N‐EA05/MG11, N° 047N‐EA05/MG11, N° 083N‐A03/MG05, N° 206 MINENVEF/SG/DGEF/DPB/SCBLF, N° 0324N/EA12/MG03, N° 100 L\fEF/SG/DGEF/DADF/SCBF, N° 0379N/EA11/MG02, N° 200N/EA05/MG02). Authorization for export was provided by the Director of Natural Resources.

### 2.2 Gaugers

We addressed the question of whether or not the morphometric measurements performed by eleven gaugers (“measurers”) could be considered repeatable based on statistical thresholds. Eleven volunteers from three continents and six countries, who all have different levels of taxonomic training and skill, were asked to perform a pair of measurements on the same set of ant specimens with their own equipment. Eight of the volunteers are myrmecologists and three are nonmyrmecologists (two are wasp specialists and one is a dipterologist). The wide range of the observers' morphometric skills and the different levels of laboratory facilities and equipment, especially the types of microscopes used, provided an overview of morphometric reproducibility as it works in daily practice. Data belonging to gaugers appear anonymously in this paper, but in order to provide the most important information regarding their skills and their equipment's quality, gaugers are coded in triad format as follows: expertise in field, estimated total number of specimens measured in their career, and the maximum magnification of the microscope used in the present study separated by underscores (e.g., MYRM_9000_100x).

### 2.3 The morphometric character recording protocol

**FIGURE 2:**
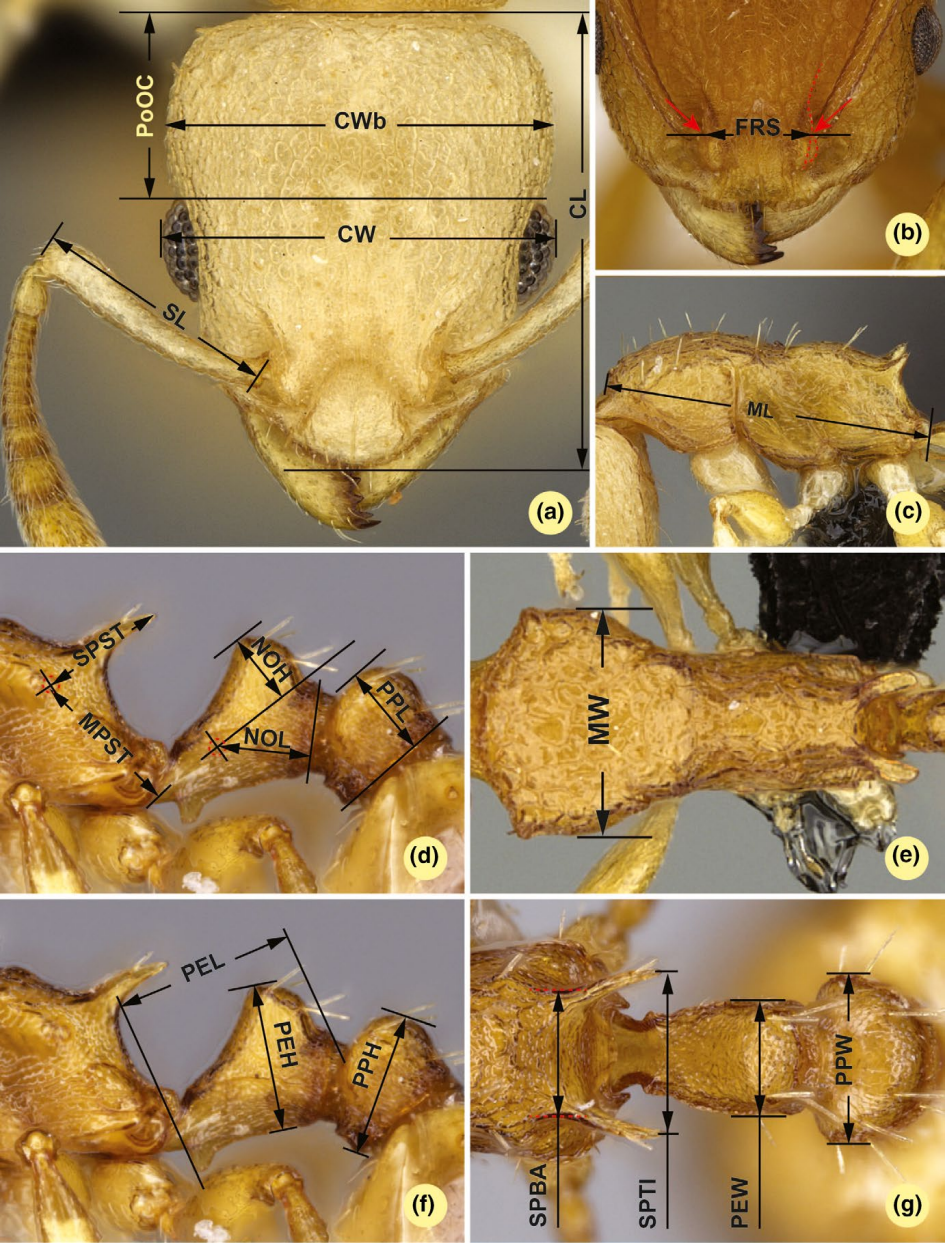
Illustrations for morphometric characters. Head in dorsal view (a) with measurement lines for CL: Head capsule length, CW: Width of head including eyes, CWb: Width of head capsule, PoOC: Postocular distance and SL: Scape length; frontal region of the head dorsum (b) with measurement lines for FRS: Frontal carinae width (red accessory lines and arrows identify the torular lamella); lateral view of mesosoma (c) with measurement line for ML: Mesosoma length; lateral view of propodeum, petiole, and postpetiole (d) with measurement lines for STPL: Propodeal spine tip erection, NOH: Maximum height of the petiolar node, NOL: Length of the petiolar node, PPL: Postpetiole length, and SPST: Spine length; dorsal view of mesosoma (e) with measurement lines for MW: mesosoma width; lateral view of propodeum, petiole, and postpetiole (f) with measurement lines for PEH: Maximum petiole height, PEL: Petiolar length, and PPH: Postpetiole height; dorsal view of propodeum, petiole, and postpetiole (g) with measurement lines for SPBA: Spine base width, SPTI: Propodeal spine tip distance, PEW: Petiole width, and PPW: Postpetiole width. Detailed verbatim trait definitions for characters are given in Table 1

Gaugers were asked to measure 21 continuous morphometric characters in each specimen twice in order to collect data for testing both intragauger error, equivalent to repeatability, and intergauger error rate, equivalent to reproducibility. Every gauger was provided the same measurement protocol, including visual and verbatim trait definitions to follow (Figure 2 and Table 1). The protocol was assembled based on an existing set of characters used in published papers (Seifert, 2006, 2018; Csősz & Fisher, 2016; Schlick‐Steiner et al., 2006; Wagner et al., 2017). In the current work, we addressed the question as to what extent random and systematic errors affect the rate of reproducibility. Therefore, all gaugers were encouraged to eliminate extraordinary differences due to gross error (occurring due to misreading, mistyping or erroneously set magnification) by comparing the values of the repeated observations.

**TABLE 1:** Verbatim trait definitions for morphometric character recording

Trait abbreviation: description	Character definition	Reference to Figure 2
CL: Head capsule length	Maximum cephalic length in median line. The head must be carefully tilted to the position with the true maximum	Figure 2a
CW: Width of head including eyes	Maximum width of the head including compound eye	Figure 2a
CWb: Width of head capsule	Maximum width of head capsule posterior of the eyes	Figure 2a
EL: Eye length	Maximum diameter of compound eye. All structurally visible ommatidiae, pigmented or not, are included	(Not illustrated)
FRS: Frontal carinae width	Distance of the frontal carinae immediately caudal of the posterior intersection points between frontal carinae and the torular lamellae. If these dorsal lamellae do not laterally surpass the frontal carinae, the deepest point of scape corner pits may be taken as reference line. These pits take up the inner corner of scape base when the scape is fully switched caudad and produce a dark triangular shadow in the lateral frontal lobes immediately posterior of the dorsal lamellae of scape joint capsule	Figure 2b
ML: Mesosoma length	Measured from caudalmost point of propodeal lobe to transition point between anterior pronotal slope and anterior pronotal shield (preferentially measured in lateral view; if the transition point is not well defined, use dorsal view and take the center of the dark‐shaded borderline between pronotal slope and pronotal shield as anterior reference point). In gynes: length from caudalmost point of propodeal lobe to the most distant point of steep anterior pronotal face	Figure 2c
STPL: Propodeal spine tip erection	Maximum distance from the center of the propodeal stigma to the margin of lateral metapleural lobe	Figure 2d
MW: mesosoma width	In workers: maximum width of the pronotum excluding the pronotal spines	Figure 2e
NOH: Maximum height of the petiolar node	Measured from the uppermost point of the petiolar node perpendicular to a reference line set from the petiolar spiracle to the imaginary midpoint of the transition between dorso‐caudal slope and dorsal profile of caudal cylinder of the petiole (Figure 1d). Do not erroneously take as reference point the dorso‐caudal corner of the helcium, which is sometimes visible. Nodal spines, if present, are excluded. If there is a dorsal plane of node (i.e., no convexity in frotal section), take care that left and right highest points of node are superimposing and use also position of setae bases for correct adjustment	Figure 2d
NOL: Length of the petiolar node	In lateral view, NOL is measured orthogonally from the reference line fitted to the margin of caudal cylinder to the center of petiolar spiracle. Take care that left and right profiles of caudal slope of node are superimposing and use also position of setae bases for correct adjustment	Figure 2d
PoOC: Postocular distance	Use a cross‐scaled ocular micrometer and adjust the head to the measuring position of CL. Caudal measuring point: median occipital margin; frontal measuring point: median head at the level of the posterior eye margin	Figure 2a
PEH: Maximum petiole height	Measured perpendicular to a ventral reference line defined as follows: the chord spanning between caudal corner of ventral petiole profile and the caudal end of the subpetiolar process. If there is a dorsal plane of node (i.e., no convexity in frontal section), take care that left and right highest points of node are superimposing and use also position of setae bases for correct adjustment	Figure 2f
PEL: Petiolar length	Diagonal petiolar length in lateral view; measured from the tip of subpetiolar process to dorso‐caudal corner of caudal cylinder. Do not erroneously take as reference point the dorso‐caudal corner of the helcium, which is sometimes visible	Figure 2f
PEW: Petiole width	Maximum width of petiole in dorsal view. Nodal spines ‐ if any ‐ are not considered	Figure 2g
PPH: Postpetiole height	Maximum height of the postpetiole in lateral view. Measured perpendicularly to a line defined by the linear section of the segment border between dorsal and ventral petiolar sclerite (Figure 1f). Take care that the lowest point of left and right part of sternites are superimposing and use also position of setae bases for correct adjustment	Figure 2f
PPL: Postpetiole length	The longest anatomical line that is perpendicular to the posterior margin of the postpetiole and is between the posterior postpetiolar margin and the anterior postpetiolar margin. Take care that the left and right part of frontal face of node are superimposing and use also position of setae bases for correct adjustment	Figure 2d
PPW: Postpetiole width	Postpetiole width. Maximum width of postpetiole in dorsal view	Figure 2g
SL: Scape length	Maximum length of the scape excluding the neck of articulatory condyle	Figure 2a
SPST: Spine length	Distance between the center of propodeal stigma and spine tip. The stigma center refers to the midpoint defined by the outer cuticular ring but not to the center of real stigma opening that may be positioned excentrically	Figure 2d
SPBA: Spine base width	The smallest distance of the lateral margins of the spines at their base. This should be measured in dorsofrontal view, since the wider parts of the ventral propodeum do not interfere with the measurement in this position. If the lateral margins of spines diverge continuously from the tip to the base, a smallest distance at base is not defined. In this case, SPBA is measured at the level of the bottom of the interspinal meniscus	Figure 2g
SPTI: Propodeal spine tip distance	Distance of propodeal spine tips in dorsal view; if spine tips are rounded or thick take the centers of spine tips as reference points	Figure 2g

Note

Abbreviations, definitions, and descriptions of morphometric characters are given. This standard protocol was followed by each gauger.

### 2.4 Data analysis

Distribution patterns of objects (i.e. specimens represented by 21 characters measured by the eleven different gaugers) were displayed in a scatterplot via principal component analysis (PCA; Venables & Ripley, 2002) using a standardization to zero mean and the variance unit (Legendre & Gallagher, 2001). A Permutational Multivariate Analysis of Variance (PERMANOVA) was performed using the Morosita index of dissimilarity with 9,999 iterations (Anderson, 2001). The applied standardization technique reduces the one site/score in comparing to the average in the PCA (Borcard et al., 2011).

Reliability depends on the magnitude of the error in the measurements to the inherent variability between subjects. These measures of variability can be expressed as standard deviations (SDs). Reliability is defined as a quadratic term of the measured values divided by the sum of the quadratic term of the measured plus the square standard deviation. It is formally described by Bartlett and Frost (2008) as (*SD* of subject's true values)^2^ = (*SD* subjects' true values)^2^ + (*SD* measurement error)^2^.

This measure of reliability is also known as intraclass correlation (ICC). If reliability is high, measurement error is small in comparison to the true differences between subjects, so that subjects can be relatively well distinguished (in terms of the quantity being measured) on the basis of the error‐prone measurements (Bartlett & Frost, 2008).

To estimate the within‐subject *SD*, we applied a one‐way analysis of variance (ANOVA) to model the data containing the repeat measurements made on subjects. In addition, we also tested the effect of the gaugers' expertise and their equipment's performance on the accuracy of ICC estimation by using Spearman's rank correlation. The analyses were carried out in R 3.6.2 (R Core Team, 2019) by using the “Vegan” package (version 2.5‐6; Oksanen et al., 2019) for PCA and PERMANOVA and “car package” (version 3.0‐7; Fox & Weisberg, 2019). Repeatability was calculated for each gauger respectively in order to assess whether the gauger's skills or equipment quality played major roles in measurement consistency.

## 3 RESULTS

### 3.1 Agreement in classification between gaugers

**FIGURE 3:**
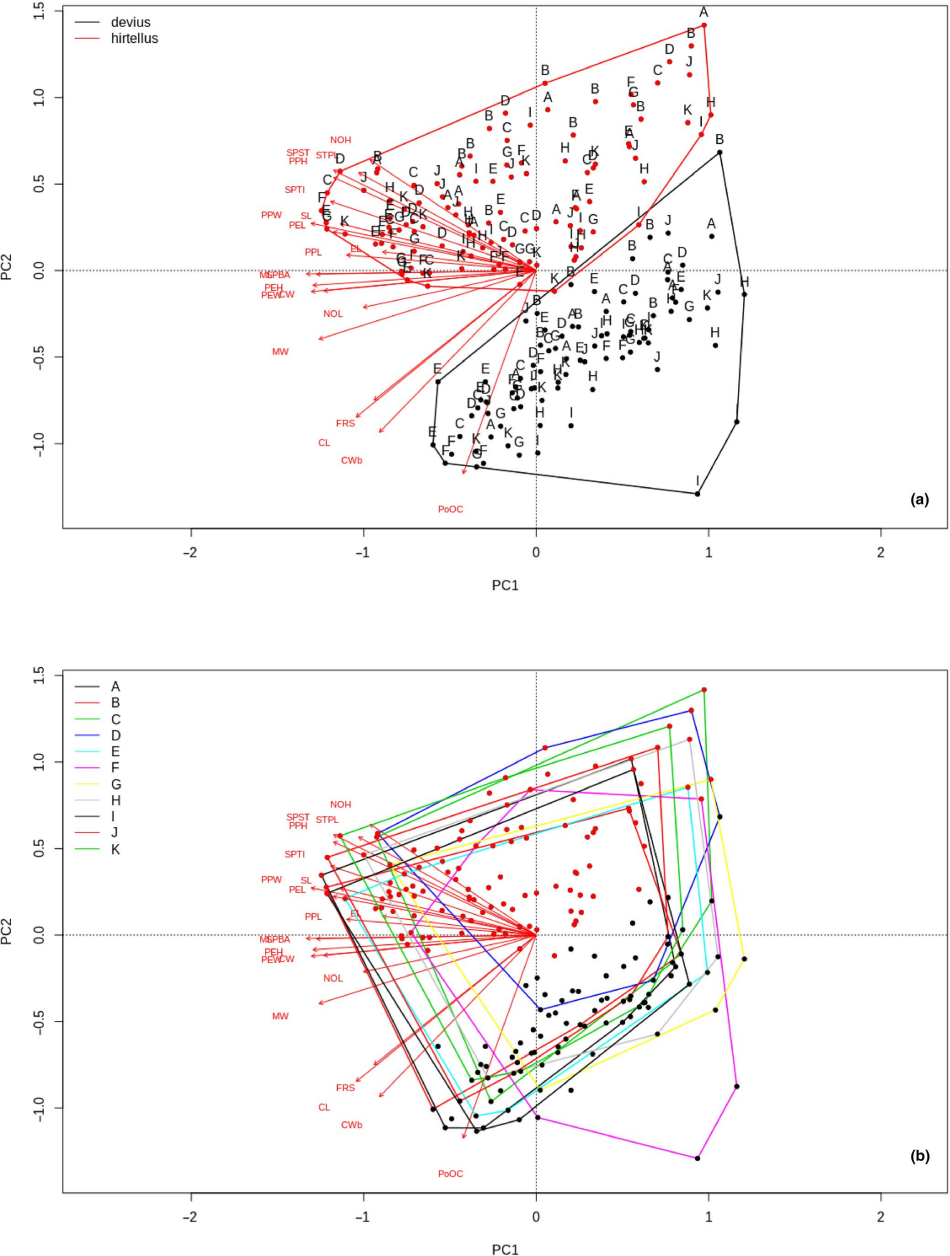
Ordination biplot for principal component analysis based on (a) distribution of observations by species identity and (b) distribution of observations by gaugers. Black and red dots represent repeated observations on the same objects, while black dots represent *Nesomyrmex devius*, and red dots represent *Nesomyrmex hirtellus*. Convex hulls for spatial distribution of observations within morphospace represent (a) species and (b) gaugers. Descriptions for abbreviations of morphometric characters (red letters) are as follows: CL: Head capsule length, CW: Width of head including eyes, CWb: Width of head capsule, FRS: Frontal carinae width ML: Mesosoma length; MW: mesosoma width; NOH: Maximum height of the petiolar node, NOL: Length of the petiolar node, PEH: Maximum petiole height, PEL: Petiolar length, PEW: Petiole width, PoOC: Postocular distance, PPH: Postpetiole height; PPL: Postpetiole length, PPW: Postpetiole width, SL: Scape length, SPBA: Spine base width, SPST: Spine length, SPTI: Propodeal spine tip distance, STPL: Propodeal spine tip erection. Gauger alphabet codes (B) in triad format: A: MYRM_9000_100x, B: DIPT_0_100x, C: MYRM_5000_288x, D: MYRM_60000_360x, E: MYRM_500_50x, F: MYRM_500_50x, G: MYRM_450_50x, H: WASP_1000_230x, I: WASP_0_230x, J: MYRM_300_100x, K: MYRM_300_100x. Compositional differences between treatments expressed as the results of the PERMANOVA (coefficient of determination, F and p values, details in the text)

The classification of the 18 pairs of independent observations made by eleven gaugers was successful for the two taxa according to the cumulative PCA analysis that involved all gaugers' observations in the same analysis. Each gauger arrived at the two‐species hypothesis with only two misidentified cases (<1%) out of the total 198 observations (Figure 3a); a single misclassification appeared in two different gaugers respectively. The results of the PCA revealed that the species identity was responsible for the differences based on the morphological traits, and was not ascribed to gauger effect (Figure 3b). The PCA results were based on the inertia 5,489, and the variance explained by the 1st axis was 62.59%, while the variance for the 2nd axis was 10.32%; thus, the overall variance explained by the first two axes was 72.91%. These patterns were also revealed by the PERMANOVA performed using the Morosita index of dissimilarity with 9,999 iteration, where the gaugers were shown to have no significant effect on the species identity based on the measured morphological traits (*R* ^2^ = 0.69, *p* = 0.58).

### 3.2 Reproducibility (intergauger agreement)

The Intraclass Correlation Coefficients (ICCs) indicated that the reproducibility of the examined 21 morphometric characters varied between *R* = 0.471 and *R* = 0.872 (mean *R* = 0.690) when the intergauger agreement was considered across the 11 gaugers (Table 2). Five morphometric traits (EL, FRS, NOL, PoOC, PPL) were found to be slightly reproducible, with intraclass correlation coefficient (*R*) scores between 0.471 and 0.529 (Table 2). These scores belong to physically smaller traits in the observed character pool, hence we examined to what extent absolute character size affects the reproducibility. The general linear model returned no significant correlation (*R* = 0.362, *p* = 0.107) between the trait size and ICC scores.

**TABLE 2:** Intraclass correlation coefficients (*R*), upper and lower bounds, number of cases (*n*) and average trait sizes are given for each observed characters

Character	*R*	Lower bound	Upper bound	*n*	Average trait size (µm)
CL	0.8113	0.7050	0.9176	18	648.45
PoOC	0.5292	0.3511	0.7074	18	250.95
CW	0.8073	0.6993	0.9154	18	565.60
CWb	0.6278	0.4629	0.7927	18	540.93
FRS	0.5259	0.3476	0.7043	18	243.82
SL	0.6826	0.5306	0.8346	18	387.69
EL	0.5116	0.3324	0.6907	18	155.03
MW	0.7970	0.6846	0.9095	18	407.84
PEW	0.8434	0.7519	0.9349	18	264.86
PPW	0.7836	0.6655	0.9016	18	300.48
SPBA	0.8724	0.7955	0.9494	18	223.93
SPTI	0.7864	0.6695	0.9032	18	265.74
ML	0.7561	0.6275	0.8847	18	762.78
PEL	0.5951	0.4244	0.7658	18	330.85
NOL	0.5087	0.3294	0.6880	18	180.08
STPL	0.6159	0.4487	0.7831	18	243.49
PEH	0.7283	0.5900	0.8666	18	249.68
NOH	0.6140	0.4465	0.7815	18	154.87
PPH	0.8169	0.7131	0.9207	18	228.55
SPST	0.8013	0.6907	0.9120	18	217.51
PPL	0.4709	0.2909	0.6508	18	185.64

Note

Descriptions for abbreviations of morphometric characters are as follows: CL: Head capsule length, CW: Width of head including eyes, CWb: Width of head capsule, FRS: Frontal carinae width ML: Mesosoma length; MW: mesosoma width; NOH: Maximum height of the petiolar node, NOL: Length of the petiolar node, PEH: Maximum petiole height, PEL: Petiolar length, PEW: Petiole width, PoOC: Postocular distance, PPH: Postpetiole height; PPL: Postpetiole length, PPW: Postpetiole width, SL: Scape length, SPBA: Spine base width, SPST: Spine length, SPTI: Propodeal spine tip distance, STPL: Propodeal spine tip erection.

### 3.3 Repeatability (intragauger agreement)

A geometric mean of intraclass coefficients was calculated for every gauger in order to evaluate their personal performance in association with their skills and equipment quality. The calculated average measure of intraclass correlation coefficients of different gaugers ranged from *R* = 0.7840 to *R* = 0.9897 (Table 3). The nonparametric Spearman's Rank correlation revealed a significant correlation between ICC scores and gaugers' morphometric skills, represented by the estimated number of individuals measured in their personal career (*n* = 11, *R* = 0.70, *t* = 2.94, *p* = 0.016) and a nonsignificant association between the repeatability parameters and the maximum magnification of the microscope (*n* = 11, *R* = 0.56, *t* = 2.03, *p* = 0.073) applied by the gauger.

**TABLE 3:** Repeatability scores (*R*) calculated for gaugers

No.	Gauger code	ICC (*R*)	Expertise	Magnification	Field
1	MYRM_9000_100x	0.93645	9,000	100	Myrmecologist
2	DIPT_0_100x	0.817519	0	100	Dipterologist
3	MYRM_60000_360x	0.989691	60,000	360	Myrmecologist
4	MYRM_5000_288x	0.971863	5,000	288	Myrmecologist
5	MYRM_500_50x	0.831945	500	50	Myrmecologist
6	MYRM_450_50x	0.903117	450	50	Myrmecologist
7	MYRM_500_50x	0.816274	500	50	Myrmecologist
8	WASP_1000_230x	0.884679	1,000	230	Parasitic wasps
9	WASP_0_230x	0.856756	0	230	Parasitic wasps
10	MYRM_300_100x	0.784024	300	100	Myrmecologist
11	MYRM_300_100x	0.87304	300	100	Myrmecologist

Note

Gauger information in the table follows this format: experience in an insect group, estimated number of individuals measured in a career, maximum magnification of the microscope used, separated by underscores. ICC: intraclass correlation coefficient calculated from the repeated measurements. The gaugers are aligned according to the sequence of their contribution. Gauger alphabet codes in triad format: A: MYRM_9000_100x, B: DIPT_0_100x, C: MYRM_5000_288x, D: MYRM_60000_360x, E: MYRM_500_50x, F: MYRM_500_50x, G: MYRM_450_50x, H: WASP_1000_230x, I: WASP_0_230x, J: MYRM_300_100x, K: MYRM_300_100x.

## 4 DISCUSSION

Morphometric characters proved reproducible in terms of intergauger agreement. The eleven gaugers successfully arrived at the same two‐species conclusion despite a great variety of morphometric skills and microscopic equipment of differing quality. The PERMANOVA test revealed no significant gauger effect on the species identity (*R* ^2^ = 0.69, *p* = 0.58). The ratio of misidentifications on specimen level over all gaugers was only 1.0% within a total of 198 determinations. The nonparametric Spearman's Rank correlation revealed that gauger ICC scores and morphometric skills were significantly correlated, whereas repeatability parameters and maximum magnification used by the gauger were not significantly correlated. These results indicate that both observer experience and better optical resolution in microscopes reduces measurement error and increases repeatability (Table 3).

In analyzing mean intragauger agreement character‐wise, the mean ICC scores (*R*) varied between 0.471 in the least reproducible character and 0.872 in the most reproducible character. This rather low average reproducibility may have different causes. One of these may be the absolute physical size of a trait. Traits with smaller sizes tended to have lower ICC scores, but when we tested this with a Spearman's Rank correlation there was no significant correlation between trait size and ICC score. This nonsignificance may be explained by the rather large minimum trait size (155 µm) in the *Nesomyrmex* test organisms where the given differences in resolution and magnification of the optical systems did not play a major role. The situation might change dramatically if, for instance, 25‐µm long antennal segments of tiny *Plagiolepis* ants were to be measured. The solution of such a task requires measurement conditions as they were given in the gaugers MYRM_60000_360x and MYRM_5000_288x.

If mean trait size does not contribute much to the rather low ICC scores in the present study, these data are probably better explained by a combination of ten error sources as they were specified for stereomicroscopy by Seifert (2002). It is impossible to analyze which of these caused major disturbances in this study. All observers received verbal and picture‐assisted character definitions (see Figure 2 and Table 1) but were given no further advice or protocols on how to minimize stereomicroscopic measuring errors. First, whether all observers avoided the parallax error is unknown. Second, whether all observers used an X‐Y‐Z‐stage for spatial positioning of specimens (see Figure 1 in Seifert, 2002) and which position stability this stage had are also unknown. In spatial positioning, it is important to place the two endpoints of a measurement in the same visual plane, which is more accurate the lower the depth of focus or the higher the magnification of the optical system. Third, the performance and reliability (e.g., ratchet‐step error) of the zoom microscopes used by gaugers in this study are unknown. Fourth, it is unknown how the observers made their readings (by one tenth of a graduation mark, by entire graduation marks, by digital read‐out systems, etc.). A fifth important error source is observer‐specific, ambiguous translation of character definitions. These factors highlight the importance of presenting unambiguous character definitions and proposing accurate measurement procedures (see supplementary file SI4, the measuring protocol of the most advanced observer).

To conclude, besides the above‐mentioned uncertainties that are common in regular practice in insect taxonomic research, morphometry has proven reproducible in our test setting. The best morphology, we believe, may be done through multimodal means, such as combining multiple microscopic and morphometric methods (e.g., Richter et al., 2019; Sarnat et al., 2019; Hita‐Garcia et al., 2019; Boudinot, 2019; Keklikoglou et al., 2019; Braga et al., 2019). Given the same size range of measured traits, the same range of observers' skill, and the same range of equipment, we expect the same reproducibility for other groups of arthropods, provided these have a similar exoskeleton stability and that specimens belong to a comparable developmental stage. Apart from this, we encourage research teams to replicate this study with taxa of different size classes, such as with tiny parasitic wasps and larger grasshoppers or crickets. The requirements for equipment will change, but we are keen to know if the basic conclusions prove comparable to our results with *Nesomyrmex* ants.

## CONFLICT OF INTEREST

The authors declare no competing financial and nonfinancial interests.

## AUTHOR CONTRIBUTIONS

**Sándor Csősz:** Conceptualization (equal); data curation (equal); formal analysis (equal); investigation (equal); methodology (equal); project administration (equal); supervision (equal); validation (equal); visualization (equal); writing – original draft (equal); writing – review & editing (equal). **Bernhard Seifert:** Conceptualization (equal); investigation (equal); methodology (equal); writing – original draft (equal). **István Mikó:** Conceptualization (equal); investigation (equal); writing – original draft (equal). **Brendon E. Boudinot:** Investigation (equal); writing – original draft (equal). **Marek Borowiec:** Investigation (equal). **Brian L. Fisher:** Conceptualization (equal); project administration (equal); writing – original draft (equal). **Matthew Prebus:** Investigation (equal). **Jayanthi Puniamoorthy:** Investigation (equal). **Jean‐Claude Rakotonirina:** Investigation (equal). **Nicole Rasoamanana:** Investigation (equal). **Roland Schultz:** Investigation (equal). **Carolyn Trietsch:** Conceptualization (equal); writing – original draft (equal). **Jonah M. Ulmer:** Investigation (equal). **Zoltán Elek:** Conceptualization (equal); formal analysis (equal); validation (equal); visualization (equal); writing – original draft (equal).

## Supporting information

Supplementary Material

Supplementary Material

## Data Availability

Data available from the Dryad Digital Repository https://doi.org/10.5061/dryad.q83bk3jfq.
