# Supplementary material for: Insect morphometry is reproducible under average investigation standards
Source: Ecol Evol. 2020 Dec 8;11(1):547–59. doi: 10.1002/ece3.7075 (PMC7790639; doi:10.1002/ece3.7075)
Supplement: Supplementary file 1 — Supplementary Material [file ECE3-11-547-s001.DOC]

**4 January 2016, *Nesomyrmex* reproducibility project: Bernhard Seifert**

**Equipment**

(a) pin-holding stage, permitting full rotations around X, Y, and Z axes

(b) Leica M165C with a 2.0 planapochromatic objective (resolution 1050 lines/mm) and a cross-scaled ocular micrometer with 120 graduation marks ranging over 52 % of the visual field. To avoid the parallax error, its measuring line was constantly kept vertical within the visual field.

(c) Schott KL 1500 cold–light source with two flexible, focally mounted light–cables, providing 30°–inclined light from variable directions A Schott KL 2500 LCD cold–light source in combination with a Leica coaxial polarized–light illuminator

(d) In order t to accelerate data recording and to reduce input errors, I wrote an input program in dBASE. It reduces the recording time by about five minutes per specimen.

**Procedures**

No manipulations of the position of body parts were done. If structures were concealed (e.g., EL by scape, NOH by mounting or hind leg), I tried to find a suboptimal measuring position or resigned. The only manipulation was removal of dust particles over a measuring point.

No *post* *hoc* corrections of any measurement was allowed except for strong reading errors (e.g., if 105 instead of 95 graduation marks were read)

The 2^nd^ (repeated) measurement was done after all 19 other specimens had been measured. In other words, there were at least two working days between 1^st^ and 2^nd^ measurement.

The ratchet steps were fixed always moving from a larger to the smaller magnification and the precise ratched position was tested by very gently shaking the zoom knob.

The characters were measured at the following magnifications

CL, PoOc_L, PoOc_R, CW, CWb (designated as CW3 in my file) 150-189x

SL_L, SL_R, MW 240x (rarely 300x)

PPW, PEW, SPTI, SPBA, SPST_L, SPST_R, STPL_L, STPL_R, PEL, PEH, PPH 360x

PPL, NOL_L, NOL_R, NOH_L, NOH_R, EL_L, EL_R, FRS 360x

ML 120x (rarely 150x)

“_L” and “_R” refer to left and right body part.

“Engageable zoom notches” “ratchet steps”

No ratchet-step error occurs at magnifications at 360x. The mean ratchet-step error at magnifications between 120x and 300x was determined as 0.14% by object micrometer measurements. 14 unique and 7 bilateral measurements were performed. This gives a total of 14+7*2 = 28 measurements. Bilateral measurements were done in PoOc, SL, SPST, STPL, NOL, NOH, EL.

Due to asymmetry of spiracle positions, it is important to measure SPST, STPL, NOL and NOH

bilaterally – the "E.O.Wilson mode" of mounting does not always allow this.

In characters with difficult spatial positioning (PEH, PPH, PPL, NOL, NOH), the best compromise of any available information was chosen for correct spatial adjustment. This information also included the position of setae bases.

**Comments on specimens**

Specimen 14: The right spine is broken off. A substitute value for SPTI was constructed assuming a left-right symmetry – i.e.. the doubled left-side deviation of spine tip from spine base was added to SPBA.

Specimen 15: PEL is not measurable both from left and right viewing position. I calculated a *N.* *devius*-specific substitute value using regression functions with the two most correlated characters.

**Results and Conclusions**

The measuring time per specimen was 44.0 ± 3.0 minutes for 28 measurements. I worked with uttermost care. The mean measuring error over 21 characters was 0.26%, ranging from 0.10% in CL to 0.49% in PPL.

The file “Correlations_Error.xls” shows Pearson’s correlation coefficient and the mean measuring errors.

All 20 characters are sufficiently reproducible if one carefully considers the character definitions (with my supplementary advice) and the error sources explained by Seifert (2002). Measuring errors have no influence on classification success. The PCA of CS and 19 shape variables shows a very close position of 1^st^ and 2^nd^ measurement. If specimen numbers are missing in the plot (No 7, 14) they are concealed through superimposing by other numbers:


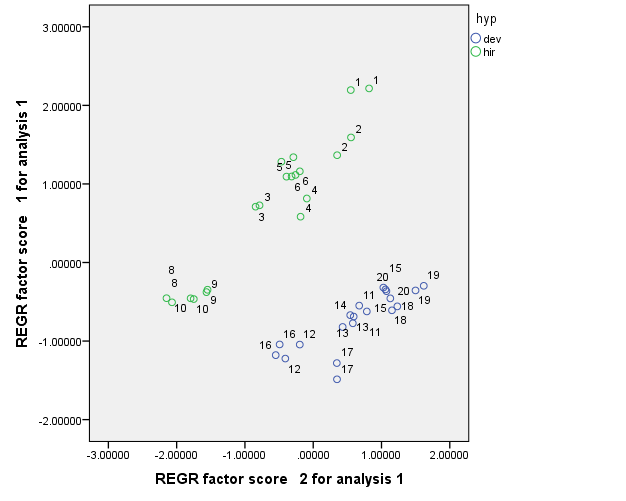


*N. devius* and *hirtellus* are similar but no cryptic species. There are two non-overlapping shape characters and it should be possible to build up a subjective recognition by simple eye inspection. See also the table overleaf.

|  | ***devius***  (i=20) | ***hirtellus***  (i=20) |
| --- | --- | --- |
| CS | 604.6 ± 11.4  [583.6,622.1] | 609.9 ± 17.0  [567.8,624.9] |
| CL/CW | 1.168 ± 0.011  [1.152,1.186] | 1.134 ± 0.012  [1.116,1.158] |
| SL/CS | 0.629 ± 0.006  [0.618,0.638] | 0.654 ± 0.008  [0.642,0.670] |
| PoOc/CL | 0.396 ± 0.006  [0.383,0.401] | 0.381 ± 0.006  [0.370,0.389] |
| EL/CS | 0.256 ± 0.005  [0.248,0.265] | 0.258 ± 0.008  [0.246,0.270] |
| FRS/CS | 0.405 ± 0.009  [0.391,0.421] | 0.395 ± 0.007  [0.384,0.410] |
| MW/CS | 0.674 ± 0.012  [0.644,0.692] | 0.673 ± 0.018  [0.646,0.710] |
| ML/CS | 1.246 ± 0.013  [1.225,1.267] | 1.285 ± 0.025  [1.247,1.330] |
| SPBA/CS | 0.361 ± 0.015  [0.338,0.387] | 0.376 ± 0.022  [0.345,0.411] |
| SPTI/CS | 0.423 ± 0.011  [0.398,0.437] | 0.454 ± 0.023  [0.429,0.500] |
| SPST/CS | 0.337 ± 0.008  [0.323,0.355] | 0.371 ± 0.007  [0.357,0.384] |
| STPL/CS | 0.398 ± 0.009  [0.382,0.411] | 0.425 ± 0.015  [0.398,0.444] |
| PEW/CS | 0.438 ± 0.013  [0.407,0.451] | 0.444 ± 0.019  [0.416,0.487] |
| PPW/CS | 0.494 ± 0.010  [0.474,0.507] | 0.510 ± 0.023  [0.478,0.553] |
| PEH/CS | 0.405 ± 0.006  [0.390,0.415] | 0.415 ± 0.016  [0.385,0.437] |
| PEL/CS | 0.550 ± 0.013  [0.530,0.580] | 0.571 ± 0.017  [0.546,0.603] |
| NOL/CS | 0.286 ± 0.014  [0.270,0.308] | 0.297 ± 0.007  [0.286,0.309] |
| NOH/CS | 0.237 ± 0.011  [0.217,0.257] | 0.272 ± 0.011  [0.254,0.292] |
| PPL/CS | 0.292 ± 0.007  [0.279,0.302] | 0.303 ± 0.008  [0.289,0.316] |
| PPH/CS | 0.363 ± 0.009  [0.344,0.376] | 0.388 ± 0.019  [0.360,0.420] |
